# Editorial: “How to Improve Cord Blood Transplantation: By Enhancing Cell Counts or Engraftment?”

**DOI:** 10.3389/fmed.2016.00020

**Published:** 2016-05-13

**Authors:** Meral Beksac

**Affiliations:** ^1^Department of Hematology and Cord Blood Bank, Ankara University, Ankara, Turkey

**Keywords:** cord blood transplantation, engraftment, expansion, cellular therapy, natural killer cells

**The Editorial on the Research Topic**

**How to Improve Cord Blood Transplantation: By Enhancing Cell Counts or Engraftment?**

Among many sources of hematopoietic stem cells (HSCs), cord blood (CB) belongs to the earliest stages of life, rich of stem cells with longest telomeres and capable of the longest life-span following transplantation (1). Thus, CB has many features resembling fetal or even embryonic stem cells. Although stem cell content of CB was known for a long time the first and also successful human cord blood transplantation (CBT) was made possible in 1988. So far, more than 30,000 CBTs have been performed worldwide. Although the initial experience began with CBTs from human leukocyte antigen (HLA)-matched sibling CB, later unrelated CB has become the major source (Beksac). Ease of procurement and lack of donor attrition, with the ability to process and store the donor cells long term make CB as an attractive HSC source. Importantly, high proliferative potential of the immature HSCs allows one log less use of cells compared to bone marrow (BM) or peripheral blood stem cells. Furthermore, due to the immune nature of CB, it provides an excellent alternative source of HSC for patients lacking HLA-matched donor. Up to two out of six HLA antigen mismatches are easily acceptable.

Cord blood is either donated to public CB banks for use by any patient worldwide for whom it is a match or stored in a private bank for potential autologous or family use. In this mini-review, there is a chapter by Armitage on the regulations, standards, and accreditations schemes currently available nationally and internationally for public and private CB banking. Currently, most of private banking is not regulated as strictly as public CB banks. Regulations and standards were initially developed to address the public banking. However, the importance of quality concept and accreditation is gradually becoming recognized by autologous and hybrid banks. Greater success rate of cords coming from accredited CBBs is also a scientifically recognized finding (2). To ensure success and the true realization of the full potential of CB, whether for autologous or allogeneic use, it is essential that each and every product meets high-quality, international standards. Authorities, such as AABB and FACT/NETCORD, aim to ensure quality among CBBs worldwide.

In spite of all the above listed advantages, there are limitations, such as low cell dose, which is associated with prolonged time to engraftment, risk of graft rejection, infections, and treatment-related mortality. To increase the cell dose, a variety of *ex vivo* expansion techniques have been developed. In this mini-review, there is a chapter written by the group led by Shpall, MD Anderson Cancer Center. They will discuss their pioneering experience of expanding CB cells with mesenchymal progenitor cells (Mehta et al.) and other methods such as use of HPC-differentiation blockers, i.e., nicotinamide analogs, copper chelators, inducing constitutive Notch signaling, or an aryl hydrocarbon receptor antagonist (StemReginin1). Many of these methods lead to substantial expansions of CD34+ cells, and significantly improving time to engraftment in patients transplanted with the expanded products compared to the recipients of unmanipulated CBT.

Following allogeneic CBT, the risk of relapse and graft-versus-host disease (GVHD) are lower than what is typically observed after other graft sources with a similar degree of HLA mismatch. Natural killer (NK) cells have a well-defined role in both innate and adaptive immunity and as the first lymphocytes to reconstitute after HSCT and CBT, and they play a significant role in protection against early relapse. In this mini-review, The MD Anderson Cancer Center Group led by Rezvani will focus on the uses of CB NK cells in transplantation and adoptive immunotherapy (Mehta et al.). First, they will describe differences in the phenotype and functional characteristics of NK cells in CB compared to BM or peripheral blood. Then, they will review the obstacles arising from use of resting CB NK cells, and methods to overcome them. In addition, the current literature on killer-cell immunoglobulin-like receptor (KIR)–ligand mismatch and outcomes after CBT will be presented.

In addition to their strategies to increase the HSC content, methods to increase homing have also been developed. In this mini-review, a chapter will describe modalities such as intra-bone administration, fucosylation, CD26 inhibition, prostaglandin E2 derivative or complement 3 exposure, and SDF-1/CXCR4/CXCL-12 pathway modulations that have been experimented successfully (Beksac and Yurdakul). These *in vivo* and *in vitro* applications have yielded satisfactory results, which led to early phase clinical trials revealing faster *in vivo* engraftment.

Transplant Registry data from CIBMTR or WBMT show that approximately 9–15% of allogeneic transplants are being transplanted from CB (3, 4). Choice of CB as a stem cell source is determined by multiple factors including availability of successful institutional protocols or experience and country/population/donor-CB registry characteristics. Recently, there is a gradual decrease in the number of CBTs. Although CBs are easily accessible, high CB fees or alternative stem cell sources are limiting factors against wider use of single and double CBTs. The biggest challenge against CBT is the documented delay in myeloid and lymphoid engraftment. Nevertheless, presence of hereditary diseases within the family or lack of haploidentical family members suitable for stem cell donation makes CB an option when no matched unrelated donor is available.

In summary, recent developments described here are very promising toward overcoming hurdles and the rebirth of CBT.

## Author Contributions

The author confirms being the sole contributor of this work and approved it for publication.

## Conflict of Interest Statement

The author declares that the research was conducted in the absence of any commercial or financial relationships that could be construed as a potential conflict of interest.
